# Patients with moderate Alzheimer’s disease engage in verbal reminiscence with the support of a computer-aided program: a pilot study

**DOI:** 10.3389/fnagi.2015.00109

**Published:** 2015-06-03

**Authors:** Giulio E. Lancioni, Nirbhay N. Singh, Mark F. O’Reilly, Jeff Sigafoos, Fiora D’Amico, Gabriele Ferlisi, Floriana Denitto, Floriana De Vanna, Marta Olivetti Belardinelli

**Affiliations:** ^1^Department of Neuroscience and Sense Organs, University of BariBari, Italy; ^2^Medical College of Georgia, Georgia Regents UniversityAugusta, GA, USA; ^3^Department of Special Education, University of Texas at AustinAustin, TX, USA; ^4^Department of Educational Psychology, Victoria University of WellingtonWellington, New Zealand; ^5^S. Raffaele Medical Care CenterAlberobello, Italy; ^6^Lega F. D’Oro Research CenterOsimo, Italy; ^7^ISPE Medical Care CenterMola di Bari, Italy; ^8^Memory Drops Day CenterGiovinazzo, Italy; ^9^Department of Psychology, “Sapienza” University of RomeRome, Italy

**Keywords:** Alzheimer’s disease, reminiscence, computer-aided program, verbal cues, visual cues

## Abstract

This study focused on the assessment of a program recently developed for helping patients with moderate Alzheimer’s disease engage in computer-mediated verbal reminiscence (Lancioni et al., 2014a). Sixteen participants were involved in the study. Six of them used the original program version with the computer showing a virtual partner posing questions and providing attention and guidance. The other 10 used a slightly modified program version with the computer presenting photos and videos and providing encouragements to talk as well as attention and guidance. Participants were exposed to brief program sessions individually. The results showed that 15 participants (five of those using the first version and all of those using the second version) had a clear and lasting increase in verbal engagement/reminiscence during the intervention sessions with the program. Those 15 participants had mean percentages of intervals with verbal engagement/reminiscence below 10 during baseline and between about 45 and 75 during the intervention. The results’ implications and the need for new research were discussed.

## Introduction

Alzheimer’s disease is a neurodegenerative disorder that causes a progressive decline of the person’s general condition, which is reflected in a gradual loss of independence, with a reduction of activity, social interaction, and verbal engagement (De Leo et al., 2011; Melrose et al., 2011; Ambrose, 2012; Bernick et al., 2012; Soto et al., 2012; Spalletta et al., 2012; Wilson et al., 2012; Sikkes et al., 2013; Perri et al., 2014). While it is impossible to prevent the occurrence of the disease or to cure it successfully (i.e., eliminate its effects), a number of pharmacological and behavioral intervention strategies have been recommended to slow down the deterioration process, support adaptive performance, and improve social appearance (Giordano et al., 2010; Ferrero-Arias et al., 2011; Bharwani et al., 2012; Boller et al., 2012; Kim et al., 2012; de Vries, 2013; Schecker et al., 2013; Berk et al., 2014; Kurz and Grimmer, 2014; Tifratene et al., 2014).

Recommended behavioral intervention strategies for persons in the earlier stages of the disease have focused, among others, on assisting those persons with the: (a) performance of daily activities; (b) orientation and travel in indoor and limited outdoor areas; and (c) verbal reminiscence (Lancioni et al., 2010, 2012, 2013a,b, 2014b; Caffò et al., 2012, 2014; Crete-Nishihata et al., 2012; Serrani Azcurra, 2012; Subramaniam and Woods, 2012; Cavallo et al., 2013; Lanza et al., 2014; Singh et al., 2014; Wingbermuehle et al., 2014). In each of these areas, technology-aided programs have been developed with the aim of enabling the persons to achieve satisfactory performance independent of staff intervention (Singh et al., 2014). For example, Lancioni et al. (2009a, 2013b) and Perilli et al. (2013) have successfully assessed computer-aided programs for presenting verbal or visual instructions to help persons with mild and moderate Alzheimer’s disease perform multistep daily activities on their own. Caffò et al. (2014) and Lancioni et al. (2013a,b) have shown that persons with moderate Alzheimer’s disease can orient and travel independently to specific destinations within their living environments through the use of technology-aided programs providing auditory orientation cues. Lanza et al. (2014) have extended the assessment of orientation technology to limited outdoor spaces (i.e., a hospital campus). They showed that a portable orientation device was effective in helping participants with mild to moderate Alzheimer’s disease reach their destinations after a 15-min training on the functioning of the device. Finally, Lancioni et al. (2014a) were able to increase positive verbal engagement/reminiscence in persons with moderate Alzheimer’s disease through a computer-aided program that worked independently of staff involvement (cf., Lazar et al., 2014). That is, the participants saw a virtual partner on the computer screen who posed questions about their past experiences and provided them with positive attention and verbal guidance (prompts/encouragements).

Work within each of the aforementioned areas may be considered highly relevant for helping persons with mild and moderate Alzheimer’s disease maintain an active role with possible benefits for: (a) their overall functioning and level of satisfaction (i.e., for increasing their positive performance, improving their mood and social appearance, and possibly delaying their decline); and (b) the practical and emotional condition of staff and caregivers working with them (i.e., for providing these personnel some relief and hopefulness; Lancioni et al., 2009a,b, 2010; Woods et al., 2009, 2012; Yasuda et al., 2009; Buettner et al., 2010; Godwin et al., 2013; Lundberg, 2014; Singh et al., 2014). While a number of studies have been carried out to assess the technology-aided programs available for supporting independent activities and orientation/travel, only limited evidence is available with regard to the program for supporting independent (i.e., computer-mediated) verbal engagement/reminiscence.

This study focused on the assessment of such a verbal engagement/reminiscence program to gather new evidence as to its practical consistency, that is, its ability to support verbal reminiscence independent of staff or therapist’s guidance (Barlow et al., 2009; Lazar et al., 2014). For the assessment, we used a program version identical to that previously employed by Lancioni et al. (2014a) (i.e., with the computer showing a virtual partner; see above) and a modified version. The latter did not include the virtual partner. It relied on the computer presenting photos and video clips of the participants or of relevant people/events and places (cf., Astell et al., 2010a), and providing a verbal description of and an encouragement to talk about them. The use of the two versions was thought to be important to ascertain whether the program would still work regardless of arrangement variations that one might adopt for practical reasons related to the intervention context or participants. The hypothesis was that both versions could be effective in promoting participants’ independent (i.e., computer-mediated) verbal engagement/reminiscence. Sixteen participants with moderate Alzheimer’s disease were involved in the study. Six of them (i.e., the first six enrolled in the study) used the program version reported by Lancioni et al. (2014a) while the other 10 used the modified version.

## Method

### Participants

The first six participants enrolled in the study (Participants 1–6; see Table 1), who used the original program version, included three females and three males aged 77–93 (*M* = 84) years. The other 10 participants (Participants 7–16; see Table 1), who used the second program version, included eight females and two males aged 70–92 (*M* = 82) years. All participants were fairly quiet and generally silent within their context but were capable of responding to verbal questions and encouragements, and of watching photos and videos and talking about them. They had a diagnosis of moderate Alzheimer’s disease with scores on the Mini Mental State Examination (Folstein et al., 1975) ranging from 11 to 17 (*M* = 15) for the first six participants and from 12 to 18 (*M* = 14) for the last 10 participants (see Table 1).

**Table 1:** **Participants’ characteristics**.

Participant	Sex	Age	MMSE
1	M	83	14
2	F	93	11
3	M	80	17
4	F	77	16
5	F	85	16
6	M	84	15
7	F	81	12
8	F	88	14
9	M	70	18
10	F	92	13
11	F	91	12
12	F	82	12
13	F	85	14
14	F	84	18
15	F	72	13
16	M	75	17

*MMSE stands for Mini Mental State Examination*.

The participants attended centers for persons with Alzheimer’s disease and other dementias in which they were involved in self-care (e.g., grooming) and leisure (e.g., music listening) activities or other simple occupational activities. In spite of the emphasis on positive activity engagement, they could spend various periods of the day sitting with persons in similar conditions (i.e., attending the same context) in a fairly passive manner and with marginal staff intervention/attention. Those periods of inactivity and virtual silence were considered detrimental for them and an intervention strategy to foster their alertness and involvement was viewed as desirable. Using a simple computer-aided program to help the participants reminisce events of their life and increase their positive verbal engagement seemed a reasonable and affordable intervention strategy. Staff and families, who had seen preliminary versions of such program, supported it. Participants were thought to be comfortable about it and, possibly to enjoy it, based on the information available about them (i.e., their ability to respond to questions and encouragements and to watch and talk about photos and videos). Families had also provided formal consent for the participants’ involvement in the study, which had been approved by the Ethics Committee of the Walden Technology S.r.l., Rome, Italy.

### Technology and Questions/Topics for the First Program Version

The technology for the first program version matched that used by Lancioni et al. (2014a) and involved a computer system with screen and sound amplifier, a microswitch, and specific software. During intervention sessions, the participant: (a) sat in front of the computer screen with the microswitch (i.e., a push button); and (b) was shown video-recorded sequences of a virtual partner (i.e., a woman matching typical caregiving figures) who greeted him or her, presented questions (relevant topics) for engagement/reminiscence, and provided positive attention, and guidance (i.e., prompts/encouragements). As in Lancioni et al. (2014a), the questions posed by the virtual partner on the screen covered several topics per participant (e.g., cooking and husband’s eating, work, church and prayers, children, house, neighbors, music and dance). For each topic, different sets of questions were available. Topics and questions varied across sessions.

A session started with the virtual partner greeting the patient and posing the first question. For example, she could ask about the participant’s active role in the local church. Assuming that the participant would respond to the question, the virtual partner displayed positive nodding (possibly accompanied by approving vocal sounds) and, after about 15 s, verbalized a positive comment (e.g., you did so much for your church!). Then, the partner encouraged the participant with a phrase such as “Let’s continue, press the push-button” (i.e., the microswitch). If the participant pressed the push-button, the partner showed animation/smiles and presented the second question (e.g., about the participant’s singing in church). Following the question, the partner performed as described above and after about 15 s made a positive statement (e.g., I am sure it was lovely!). If the participant did not press the push-button independently, the partner encouraged such a response and then posed the third question (e.g., about the ceremonies or songs the participant liked the most). The procedure continued the same way with approval and new questions until 5 min had elapsed and the session was ended. If a participant did not respond to the partner’s encouragement to press the push-button, the encouragement would be repeated at intervals of approximately 15 s. The encouragement could also be programmed at longer intervals and/or uttered at a relatively low intensity if the participant tended to talk for rather long periods of time in relation to the questions presented.

### Technology and Visual and Verbal Cues for the Second Program Version

The second program version replaced the appearance of the virtual partner and the questions with photos or 2- to 4-s video clips of the participant him- or herself in special circumstances (e.g., children’s weddings or other celebrations) or of relevant people/events and places. The computer accompanied the photos and the videos (the last frame of which remained on view like the photos) with a brief verbal description of and an encouragement to the participant to talk about them. Assuming that the participant would talk about what was on view, the computer produced approving vocal sounds, which were then followed by a positive comment (i.e., as in the first version). An encouragement to press the microswitch to talk about something else occurred after 20 s or more (i.e., if the participant had not activated the microswitch independently). Microswitch activation caused the previous photo or the last frame of the previous video to be replaced by a new photo or video accompanied by a verbal description and an encouragement to the participant to talk about it. In case of no microswitch activation, the computer would provide a new encouragement at intervals of about 20 s. Between 40 and 50 photos and videos were available for each participant. The use of those visual cues (and accompanying verbal cues) was rotated across sessions.

### Setting, Sessions, and Data Recording

Computer-aided sessions as well as baseline and control sessions were carried out in a room of the centers that the patients attended. All sessions lasted 5 min. Typically, two or three computer-aided sessions occurred per day per participant (i.e., sessions were carried out on an individual basis). Control sessions were scattered through the intervention phase (see below). Sessions were video-recorded and then scored by a research assistant. A second research assistant joined in the scoring of more than 25% of the sessions to assess interrater agreement. The measures recorded were: microswitch activations and verbal engagement/reminiscence. The first measure was recorded in terms of frequency per session. The latter measure was recorded according to a time sampling procedure, using intervals of 10 s (Kazdin, 2001). Percentages of interrater agreement on the two measures (computed by dividing the smaller activation frequency by the larger one or the intervals with agreement by the total number of intervals and multiplying by 100) were within the 80–100 range, with individual means exceeding 90.

### Experimental Conditions

Each program version was introduced according to a non-concurrent multiple baseline design across participants (Barlow et al., 2009). The baseline phase included two or four sessions per participant. The following intervention phase included 80–117 (*M* = 97) sessions for the participants using the first program version and 73–122 (*M* = 99) sessions for the participants using the second program version. Differences in number of sessions were largely due to participants’ availability. Parallel to the intervention phase, the participants received 9–24 (*M* = 16) control sessions.

#### Baseline

During the baseline sessions, the participant sat in front of the computer screen, which was dark, and had the microswitch whose activation did not produce any effects.

#### Intervention

During the intervention sessions, the participant sat in front of the computer screen with the microswitch, experiencing the conditions described in the technology section regarding the first program version (Participants 1–6) or the second program version (Participants 7–16). Prior to the start of the intervention phase, each participant received five to seven practice sessions during which the research assistant guided him or her in using the microswitch and talking in response to the virtual partner’s questions or to the photos/videos and accompanying encouragements (see Technology sections).

#### Control

During the control sessions, the participant sat with other persons with dementia attending the same context, without any programmed occupation/interaction except for staff providing routine supervision.

## Results

**Figure 1:**
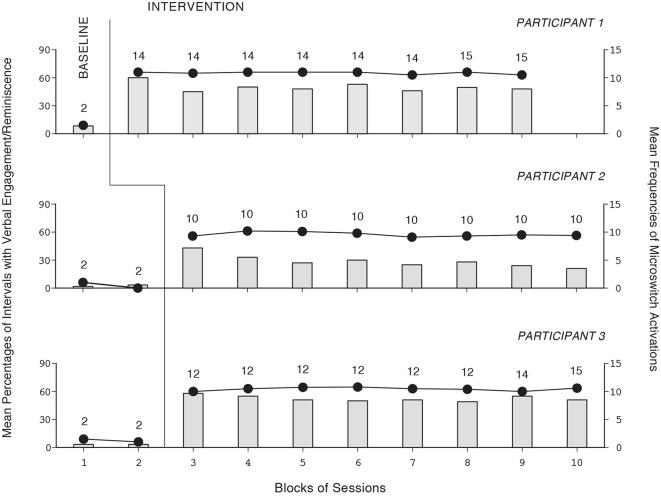
**The three panels report the data for Participants 1–3, respectively**. Bars and circles represent mean percentages of intervals with verbal engagement/reminiscence and mean frequencies of microswitch activations per session, respectively, over blocks of sessions. The number of sessions included in each block (i.e., bar-circle combination) is indicated by the numeral above it.

**Figure 2:**
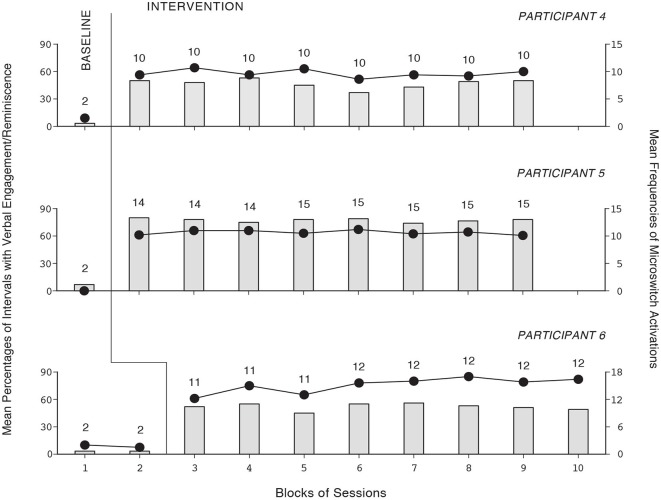
**The three panels report the data for Participants 4–6, respectively**. Data are plotted as in Figure 1.

The baseline and intervention data for the six participants using the first version of the program are summarized in Figures 1, 2. The three panels of Figure 1 report the data for Participants 1–3, respectively. The three panels of Figure 2 report the data for Participants 4–6, respectively. Bars and circles represent mean percentages of intervals with verbal engagement/reminiscence and mean frequencies of microswitch activations per session, respectively, over blocks of sessions. The number of sessions included in each block (i.e., bar-circle combination) is indicated by the numeral above it. During the baseline sessions, the participants’ mean percentages of intervals with verbal engagement/reminiscence were below 10. Their mean frequencies of microswitch activation were below two per session. During the intervention sessions, all participants showed performance improvement. Their mean percentages of intervals with verbal engagement/reminiscence ranged from near 30 (Participant 2, whose performance level was apparently declining) to about 75 (Participant 5). The participants’ mean frequencies of microswitch activation were between about 10 and 15 per session. During the control sessions (not reported in the figures), their mean percentages of intervals with verbal engagement/reminiscence were below 10.

**Figure 3:**
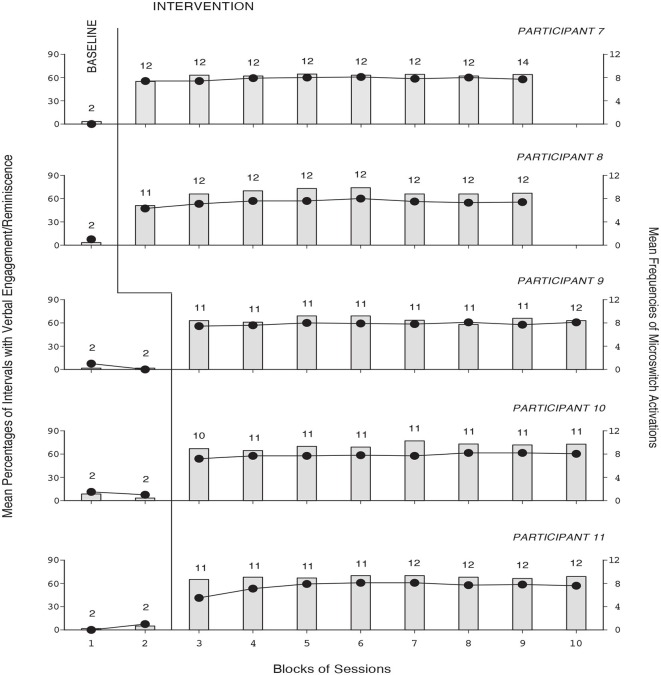
**The five panels report the data for Participants 7–11, respectively**. Data are plotted as in Figure 1.

**Figure 4:**
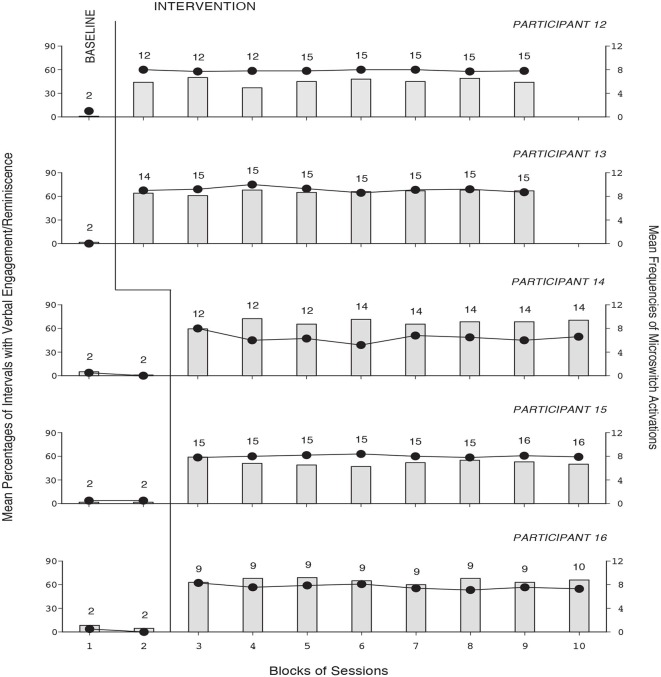
**The five panels report the data for Participants 12–16, respectively**. Data are plotted as in Figure 1.

The baseline and intervention data for the 10 participants using the second version of the program are summarized in Figures 3, 4. The five panels of Figure 3 report the data for Participants 7–11, respectively. The five panels of Figure 4 report the data for Participants 12–16, respectively. The data are plotted as in Figures 1, 2. During the baseline sessions, the participants’ performance matched that of the six participants exposed to the first version of the program. During the intervention sessions, their mean percentages of intervals with verbal engagement/reminiscence ranged from about 45 (Participant 12) to above 70 (Participant 10). Their mean frequencies of microswitch activation ranged from above six to about nine per session. During the control sessions (not reported in the figures), their mean percentages of intervals with verbal engagement/reminiscence were as in baseline.

## Discussion

The data show that 15 of the 16 participants had a clear and lasting increase in verbal engagement/reminiscence. The program was applied on an individual basis (i.e., as this seems the most appropriate approach for these participants; Chiang et al., 2010; Dahlin and Rydén, 2011; Subramaniam and Woods, 2012; Blake, 2013; Van Bogaert et al., 2013; Wingbermuehle et al., 2014). Although no comparisons were made between the two program versions, the results of this study and of the one by Lancioni et al. (2014a) suggest that both might be viable solutions for promoting independent (i.e., computer-mediated) verbal engagement/reminiscence in persons with moderate Alzheimer’s disease. In light of these still preliminary results, a few considerations might be put forward.

The verbal engagement/reminiscence exhibited by the participants during the intervention might be essentially ascribed to the use of topics (past experiences) that the participants could connect with and to the availability of verbal questions or combinations of visual and verbal cues that worked fairly adequately for them. Apparently, the two versions of the computer-aided program provided sufficient support to the participants so that they could engage in verbal reminiscence without the presence of a therapist or prompter (Kuwahara et al., 2006, 2010; Astell et al., 2010b; Lazar et al., 2014). Both program versions relied on three main intervention conditions, that is: (a) helping the participants focus their attention on the topics presented; (b) guiding them to shift their attention across various topics, thus providing them the opportunity to vary and expand their verbal engagement/reminiscence; and (c) ensuring the occurrence of positive attention/comments that could encourage the participants during their engagement and reinforce them for it (Kazdin, 2001; Bemelmans et al., 2012; Catania, 2012; Yamagami et al., 2012; Lancioni et al., 2014a).

The second program version adds a critical visual component to the conditions used in the first version. In practice, photos/videos are used to present the topic while the accompanying verbal cues and encouragements help the participant focus on the topic and start talking about it. One would expect this latter version to be as effective as the former in general. In some cases (i.e., when the participants are less attentive to verbal questions), the latter version might even have a slight advantage (cf., Zannino et al., 2010; Lancioni et al., 2012). Obviously, these early data on the two program versions can only be taken as a preliminary demonstration of their applicability and potential (Kennedy, 2005; Lundberg, 2014). Definite statements about them must be postponed until new studies have established their dependability and ascertained possible differences in their levels of impact or relations with participants’ characteristics (Kennedy, 2005; Barlow et al., 2009; Davis et al., 2012).

The differences observed among the individual levels of verbal engagement/reminiscence might have reflected the participants’ general functioning abilities, their verbal inclinations, and their tendency toward the reminiscence task. For example, the relatively low and declining performance of Participant 2 might have been due to her rather compromised and deteriorating condition that increasingly curtailed her interest in the topics presented as well as her verbal behavior and active participation (Soto et al., 2012; Spalletta et al., 2012; Wilson et al., 2012; Lancioni et al., 2014a). Careful selection of relevant topics/questions for verbal engagement/reminiscence and strongly motivating comments may help enhance the program effectiveness over time (i.e., at least until the participant’s condition seriously deteriorates) (Pierce and Cheney, 2008; Catania, 2012; Noguchi et al., 2013).

In conclusion, the results indicate that a simple program might be used profitably for supporting independent (i.e., computer-mediated) verbal engagement/reminiscence. This encouraging statement needs to be taken with caution given the still preliminary level of the data. A primary goal of new research should be to extend the assessment of the program versions to additional participants to gather extra (necessary) data to determine the solidity of the results and thus the dependability (relative effectiveness) of those versions (Kennedy, 2005; Barlow et al., 2009). New research should also pursue an upgrading of the technology used to support those versions so as to make them more effective and more easily applicable (de Joode et al., 2012; De Joode et al., 2013; Robert et al., 2013). Another research point with important practical implications could be the investigation of staff, families, and participants’ views about those program versions (e.g., about their likeableness, desirability and potential within daily contexts, and about possible ways of improving them; Callahan et al., 2008; Meiland et al., 2014; König et al., 2015).

## Conflict of Interest Statement

The authors declare that the research was conducted in the absence of any commercial or financial relationships that could be construed as a potential conflict of interest.
